# Anesthesia Management for a Patient Undergoing Pulmonary Endarterctomy without Cardiopulmonary Bypass

**DOI:** 10.21470/1678-9741-2018-0245

**Published:** 2019

**Authors:** Ayten Saracoglu, Onur Ermerak, Esra Yaman Savci Sirzai, Mustafa Yuksel, Zuhal Aykac, Bedrettin Yildizeli

**Affiliations:** 1 Department of Anesthesiology and Intensive Care, Marmara University Medical School, Istanbul, Turkey.; 2 Department of Thoracic Surgery, Marmara University Medical School, Istanbul, Turkey.

**Keywords:** Cardiopulmonary Bypass, Respiratory Distress Syndrome, Adult, Myocardial Stunning, Hypertension, Pulmonary, Endarterecomy, Heart-Lung Machine

## Abstract

Pulmonary endarterectomy is a curative procedure for chronic thromboembolic Pulmonary Hypertension. As usual, cardiopulmonary bypass circuit is required. However, there are several complications attributed to extracorporeal circulation. Hemodilution, systemic inflammatory response syndrome and leukocyte sequestration are circulation related complications. The severe forms include Acute Respiratory Distress Syndrome, Acute Lung Injury, myocardial stunning, dysfunction of the right ventricle, coagulopathy, postoperative stroke or renal dysfunction. In this case report, we aimed to give information about perioperative anesthesia and surgical management of pulmonary endarterectomy which was successfully managed without Cardiopulmonary Bypass.

**Table:** 

Abbreviations, acronyms & symbols				
CO	= Cardiac output		PEA	= Pulmonary endarterectomy
CPB	= Cardiopulmonary Bypass		PEEP	= Positive end-expiratory pressure
CTEPH	= Chronic thromboembolic pulmonary hypertension		PHT	= Pulmonary Hypertension
CVP	= Central Venous Pressure		PVR	= Pulmonary vascular resistance
ECG	= Electrocardiogram		ROS	= Reactive oxygen species
IBP	= Invasive Blood Pressure		SpO_2_	= Oxygen Saturation
NIRS	= Near Infrared Spectroscopy		TCA	= Total Circulatory Arrest
PA	= Pulmonary artery		TIVA	= Total Intravenous Anaesthesia

## INTRODUCTION

Pulmonary endarterectomy is a curative procedure for chronic thromboembolic pulmonary Hypertension (PHT), with an operative mortality rate of < 5 %^[1]^. Mortality may be reduced up to 10 % with an increase in the number of expert centers where more than 20 cases per year are performed^[2]^. Because medical treatment is palliative in these group of patients, lung transplantation is the only treatment option for pulmonary endarterectomy. Surgical procedure includes cardiopulmonary bypass (CPB) with sternotomy or lateral thoracotomy^[3]^. Deep hypothermic total circulatory arrest (TCA) is widely used^[4]^. Thus, during dissections, surgical view can be improved. In this case report, we aimed to give information about perioperative anesthesia and surgical management of pulmonary endarterectomy which was successfully managed without CPB. 

## CASE REPORT

**Fig. 1:**
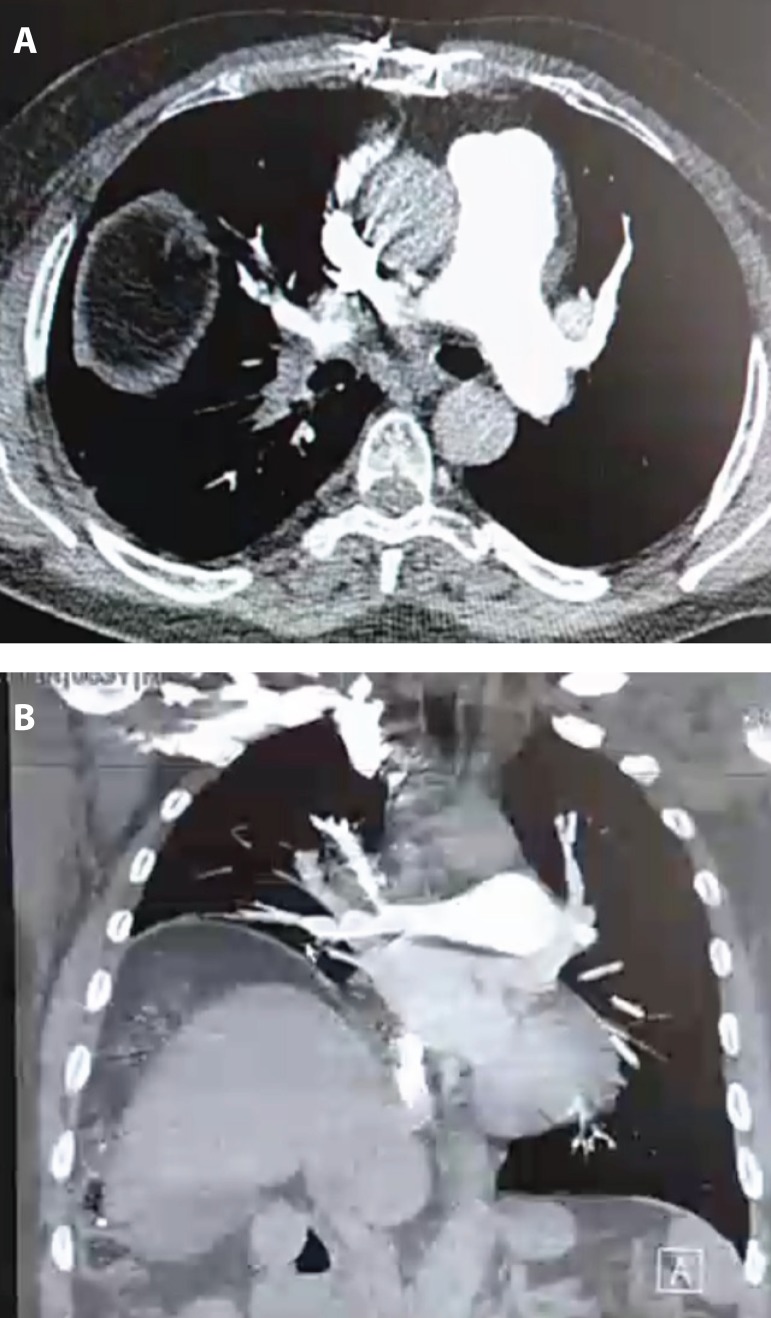
(A). CT scan showing complete thromboembolic obstruction of the right lower lobe and (B). Elevation of the right diaphragm.

A 64-year-old male with shortness of breath (New York Heart Association Class III), and elevation of right diaphragm and chronic thromboembolic pulmonary hypertension (CTEPH) for 1-year period was referred for pulmonary endarterectomy (PEA) and diaphragmatic plication. The demographics and the preoperative evaluation results are summarized in Table 1. His medical history revealed that 8years ago the patient underwent PEA for CTEPH by our team and lumber discectomy 10 years ago. He was under warfarine and inhaled bronchodilators medication. His echocardiogram revealed normal left ventricular function with an ejection fraction of 63% but a dilated right ventricle with moderate tricuspid regurgitation. A right heart catheterization revealed slightly elevated pulmonary artery (PA) pressure of 41,4/14 mm Hg (mean, 26 mm Hg) and cardiac output (CO) of 2.9 L/min and TAPSE >15mm. Pulmonary vascular resistance (PVR) was calculated as 386 dynes/s/cm^−5^. Computed tomographic pulmonary angiography of his chest showed complete obstruction in his pulmonary vasculature on the right lower lobe consistent with chronic thromboembolic disease and elevation of right diaphragm (Figure 1). Ventilation perfusion scanning confirmed the diagnosis of CTEPH. Pulmonary endarterectomy is routinely performed under general anaesthesia through a median sternotomy and using extracorporeal circulation^[5]^. However, since our patient had previous history of PEA surgery, *i.e*. previous sternotomy and only right lower lobe disease, unilateral PEA and diaphragmatic plication was performed by right thoracotomy without cardiopulmonary bypass (CPB). 

**Table 1:** The demographics and the preoperative evaluation results.

Demographics	Male, 64 years old, 72 kg
Comorbidity	Chronic Obstructive Pulmonary Disease with the requirement of home BIPAP
History	Lumber discectomy 10 years ago, Pulmonary Endartrectomy 8 years ago,
Drugs	Warfarine and inhaled bronchodilators
Echocardiography	Ejection Fraction: 63 % Mitral and Tricuspid Valve Regurgitation: +1 Sclerotic Aortic Valve Left Atrial diameter: 44 mm TAPSE: >15 mm sPAP: 41,4 mmHg Left Ventricular Hypertrophy Left ventricular diastolic dysfunction
Preoperative examination	Mallampati II ASA II New York Heart Association (NYHA) III/IV Glasgow Coma Scale:15 Breathlessness on moderate physical exertion The lung sounds normal 6-min walk test:
ECG	Sinus rhythm
Blood count	Hematocrit: 48.2 % Hemoglobin: 16.4 g/dL White Blood Cell: 5300/mL Platelet: 339 X 109
Coagulation prophile	INR: 1.12 PT: 12.4 s aPTT: 33.7 s
Blood gase	Ph: 7.44 PCO2: 37.9 PO2: 68.1 SpO2: 96.2 HCO3: 26.4 BE: 2.3 Lac: 1.5
Electrolytes	K: 4.3 Na: 129 Ca: 4.36 Cl: 113

Following informed patient’s consent, the patient was monitorized using Electrocardiogram (ECG), invasive blood pressure (IBP), oxygen saturation (SpO_2_), central venous pressure (CVP), Near Infrared Spectroscopy (NIRS) and urine output. During the induction of anesthesia a bolus dose of propofol 2.5 mg/kg and remifentanil 0.1 mcg/kg were administered intravenously. Anesthesia was maintained with propofol 6 mg/kg/h, fentanyl 2 mcg/kg/h, 60% O_2_ and 40% air. Pressure controled ventilation was used with a Pinsp:19 mmHg, Resiratory Rate of 14/min, inspiration:expiration ratio of 1:2 and a Ppeak of 21 mmHg.

There was not any complication during the induction of anesthesia. A left double lumen tube was inserted. No bleeding occured during the initial thoracotomy. No vasopressor or inotropic agent were used during the procedure. Nitroglyserine infusion was given with inhaled Nitric Oxide. 

**Fig. 2:**
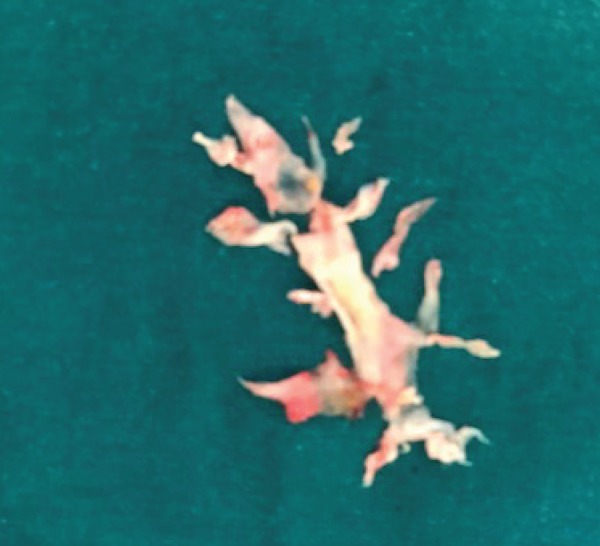
Surgical specimen of pulmonary endarterectomy.

Following right thoracotomy, right main pulmonary artery (PA) and both superior and inferior pulmonary veins are clamped. Arteriotomy was performed in the intralobar artery of the right PA and the endarterectomy specimen was circumferentially followed down to the segmental and subsegmental branches of the right lower lobe (Figure 2). Plication of the right diaphragm was performed following PEA.

The total duration for one lung ventilation was 120 min. Total cross-clamp time of pulmonary artery was 25 min. Initially, there was a bleeding amount of 800 cc. During the procedure bleeding reached to 1100 cc. Afterwards 300 ml of blood filtered through cell saver given back to the patient.

At the end of surgery, total duration of one lung ventilation was 6 hours. The arterial blood gas analysis showed a Ph of 7.33 with PCO_2_: 42.9 mmHg, PO_2_: 230 mmHg, SpO_2_: 99,6% and a BE of - 2.8. Heart rate was 78 beats/min IBP: 99/64 mmHg and the CVP was 5 mmHg. Total amount of urine output was 1500 cc and the total bleeding was 1200 cc. The amount of balanced Electrolyte Solution was 1000 cc, Colloid (Gelofusine) was 1500 cc, the Cell Saver Blood was 300 cc. Patient was transferred to the ICU postoperatively. In the ICU, Ph was 7.32, PCO_2_: 42 mmHg, PO_2_: 143 mmHg, SpO_2_: 99%, HCO_3_: 21, BE: -4.4 with the electrolytes of K: 5.8 mEq/L, Na: 135 mEq/L, Lactate: 1.3, Ca: 4.5 mEq/L, Cl: 115 mEq/L. The glucose level was 189 mg/dL with the Hb value of 14.5 g/dL and a Htc level of 45%. The patient was extubated on the 4^th^ postoperative day. The patient stayed in intensive care unit for 10 days. He was discharged after a 10-day follow-up period.

## DISCUSSION

Patient who underwent pulmonary endarterectomy due to bilateral pulmonary thromboembolism, was reoperated for right pulmonary artery lesion and was successfully managed without CPB procedure. Ever since our PEA program was started with acceptable results^[5]^, more than 500 operations have been performed over seven years (80 cases last year), with increased institutional and surgical experience resulting in our center becoming the only expert PEA center in Turkey. However, this is our first case who was operated on through right thoracotomy and without CPB.

The use of Total Intravenous Anaesthesia (TIVA), pressure controlled mechanical ventilation, and restricted fluid regimen were the pearls of successful anesthesia management.

Since deep hypothermic circulatory arrest was not applied, cerebral protection was not needed by administering mannitol, methyl prednisolone, phenytoin Na and thiopental for cerebral protection. In addition, the process of rewarming patients after TCA is also very important. Rapid heating can lead to systemic air embolisms or cerebral oxygen desaturation. Therefore, 90 to 120 minutes is required to reach a temperature of about 36.5 °C^[6]^. Due to the long aortic cross clamp time and the long hypothermic period, it may also be difficult to wean from CPB. Inotropic support may be needed during this period. Since CPB was not applied in our patient, the use of inotropic agent was not required. In addition, severe reperfusion injury may develop during weaning. This complication can occur especially in patients with right heart failure with diffuse blood clots in pulmonary artery^[7]^. During thoracotomy, one lung ventilation was applied. It has been shown that accumulation of reactive oxygen species (ROS) occur during one lung ventilation causing oxidative stress. These changes can be reduced by propofol^[8]^. In addition, propofol reduces both the amount and the duration of ROS formation in circulation. Considering these results, TIVA was preferred in our case with continuous infusion of fentanyl and propofol. Morever, unlike inhalation agents, it does not inhibit hypoxic pulmonary vasoconstriction. On the other hand, in a multicenter randomized controlled study involving 100 patients, sevoflurane and TIVA were compared; no significant difference between the two groups were found in terms of prolonged ICU stay, death, 30-day or 1-year mortality^[9]^. During thoracic surgery, one lung ventilation causes the emergence of an inflammatory response in the bronchial epithelium. Inflammatory cytokines such as TNF a, IL-1b, IL-6 , IL-8 are important chemoattractants affecting the recruitment of alveolar macrophages and neutrophils^[10]^. They also lead to inflammatory response inducing coagulation. Besides in our previous experimental study, we found that propofol caused less neutrophil infiltration in lungs, reduced free-radical-mediated lipid peroxidation and resulted in amelioration of systemic inflammation^[11]^. This was another reason why TIVA was preferred for this patient.

Furthermore, the ventilation strategy that we used intraoperatively is an important parameter that determines survival in patients. In this patient with high pulmonary artery pressure, protective lung ventilation was applied to prevent barotrauma with the use of pressure-controlled ventilator mode including the use of positive end-expiratory pressure (PEEP) and stepwise alveolar recruitment manoeuvres. Right heart performance was very susceptible being affected by positive pressure ventilation^[12]^. Thus, we also avoided to cause increased right heart pressure and right-ventricular afterload. With the use of cell salvage, unnecessary transfusion has been avoided. 

The patient underwent bilateral pulmonary endarterectomy eight years ago. This time the patient with a lesion in the right pulmonary artery did not enter the CPB because of unilateral surgery. In this rare redo case, restrictive fluid therapy was given (Table 1). Vasodilatation was caused by nitric oxide infusion, resulted in optimized perfusion in pulmonary vasculature during surgical procedure.

## CONCLUSION

In the reported case, we have demonstrated that optimizing hemodynamic management through goal directed use of blood products and preventing hypoperfusion reduces not only bleeding but also using unnecessary vasopressors and blood products during off- pump pulmonary endarterectomy. 

**Table:** 

Authors' roles & responsibilities	
AS	Substantial contributions to the conception or design of the work; or the acquisition, analysis, or interpretation of data for the work; final approval of the version to be published
OE	Substantial contributions to the conception or design of the work; or the acquisition, analysis, or interpretation of data for the work; final approval of the version to be published
EYSS	Substantial contributions to the conception or design of the work; or the acquisition, analysis, or interpretation of data for the work; final approval of the version to be published
MY	Substantial contributions to the conception or design of the work; or the acquisition, analysis, or interpretation of data for the work; final approval of the version to be published
ZA	Substantial contributions to the conception or design of the work; or the acquisition, analysis, or interpretation of data for the work; final approval of the version to be published
BY	Substantial contributions to the conception or design of the work; or the acquisition, analysis, or interpretation of data for the work; final approval of the version to be published
